# Advances in free fatty acid profiles in gestational diabetes mellitus

**DOI:** 10.1186/s12967-024-04922-4

**Published:** 2024-02-19

**Authors:** Haoyi Du, Danyang Li, Laura Monjowa Molive, Na Wu

**Affiliations:** 1 Department of Endocrinology, Shengjing Hospital of China Medical University, Shenyang, 110004 People’s Republic of China; 2 Medical Department, Shengjing Hospital of China Medical University, Liaoning Province, Shenyang, People’s Republic of China

**Keywords:** Gestational diabetes mellitus, Free fatty acids, Dietary, Fetal growth, Pregnancy outcomes

## Abstract

The morbidity of gestational diabetes mellitus (GDM) is increasing and is associated with adverse perinatal outcomes and long-term maternal and infant health. The exact mechanism underlying changes in plasma free fatty acid (FFA) profiles in patients with GDM is unknown. However, it is believed that changes in diet and lipid metabolism may play a role. Fatty acids contain many specific FFAs, and the type of FFA has different impacts on physiological processes; hence, determining changes in FFAs in individual plasma is essential. Alterations in FFA concentration or profile may facilitate insulin resistance. Additionally, some FFAs show potential to predict GDM in early pregnancy and are strongly associated with the growth and development of the fetus and occurrence of macrosomia. Here, we aimed to review changes in FFAs in women with GDM and discuss the relationship of FFAs with GDM incidence and adverse outcomes.

## Introduction

The prevalence of obesity has increased globally over the past two decades, with obesity rates exceeding 35% among women in many countries worldwide [1]. Pre-pregnancy overweight or obesity is a known risk factor for many pregnancy complications to a greater extent than gestational weight gain (GWG). The higher the pre-pregnancy maternal body mass index, the higher the risk of pregnancy complications such as pregnancy-induced hypertension (PIH) and gestational diabetes mellitus (GDM) [2, 3]. The prevalence of GDM has increased by over 35% in the last few decades and is still growing [4]. GDM pathogenesis is unclear; however, its characteristics are similar to those of type 2 diabetes mellitus, with increased insulin resistance (IR) incidence and relative insulin deficiency due to decreased β-cell function and numbers [5, 6]. Potential risk factors for GDM include pre-pregnancy overweight and obesity, age and unhealthy dietary habits [7]. Regarding dietary fat intake, higher intake of total fat saturated fatty acids (SFAs) and cholesterol in pregnant women was previously thought to be associated with an increased risk of GDM [8, 9], whereas intake of polyunsaturated fatty acids (PUFAs) and alpha-linolenic acid (ALA) was associated with a decreased risk [10]. The International Association of Diabetes and Pregnancy Study Groups (IADPSG) and World Health Organization (WHO) recommend a 75 g, 2 h oral glucose tolerance test (OGTT) for pregnant women between 24 and 28 weeks of gestation [11, 12]. The American Diabetes Association (ADA) diagnostic criteria are a fasting blood glucose level ≥ 5.1 mmol/l at the 60th minute of the OGTT or a blood glucose concentration ≥ 10 mmol/l at the 120th minute of the OGTT or a fasting blood glucose level ≥ 8.5 mmol/l at the 120th minute of the OGTT [13]. GDM is one of the most common pregnancy complications [14]. GDM is associated with poor pregnancy outcomes, abnormal glucose tolerance, and obesity in offspring [15–17]. Although preventing GDM is not possible, early diagnosis is necessary [16].

Free fatty acid (FFA) is a physiologically important energy substrate released from adipose tissue through lipolysis in response to the body's energy needs. FFA is an efficient energy store in adipocytes in the form of triacylglycerides (TAGs) and phospholipids in cell membrane components [18, 19]. Fat metabolism and carbohydrates are closely related, and alterations in FFAs may be involved in pathophysiologic processes associated with IR in GDM [20]. This review aimed to summarize the correlation between altered FFA levels and GDM to provide help in early GDM prevention and find a relationship with poor outcomes.

## Pathophysiologic process and risk factors of GDM

There is a progressive increase in insulin resistance during normal pregnancy due to an increase in circulating placental hormones, including adrenocorticotropic hormone, adrenocorticotropic hormone-releasing hormone, human placental lactogen, prolactin, estrogen, and progesterone [21–26]. Maternal obesity also promotes IR in early pregnancy, leading to increased lipolysis in the second trimester [27, 28], with the result that elevated FFA levels can further exacerbate IR by inhibiting maternal glucose uptake and stimulating hepatic gluconeogenesis [28, 29]. Increased maternal IR leads to elevated maternal postprandial glucose levels and growth-promoting FFAs [23, 27, 30]. In addition, insulin secretion increases during normal pregnancy to maintain maternal blood glucose levels [22]. In contrast, in pregnant women with GDM, insulin secretion fails to compensate for the progressive increase in insulin resistance during pregnancy, leading to the development of maternal hyperglycemia [31]. A high-fat and high-sugar diet leads to IR and β-cell dysfunction [32], and lipotoxicity caused by hypertriglyceridemia during pregnancy also contributes to pancreatic β-cell damage and further reduction in insulin secretion [33, 34]. In addition, excessive inflammatory activation may be associated with GDM, and preeclampsia possesses a similar pathophysiological process to GDM [35, 36], with pro-inflammatory mediators of arachidonic acid (AA) and linoleic acid (LA) derivatives playing an important role in the development of both [37]. The pathogenesis of GDM is similar to that of type 2 diabetes mellitus, which is characterized by increased insulin resistance and a decrease in β-cell function and number caused by relative insulin deficiency [5, 6]. There are many risk factors that contribute to the development of GDM, such as a history of GDM pregnancies [38], increasing maternal age [39], pre-pregnancy overweight or obesity [40], weight gain during pregnancy [41], and high dietary fat and low-carbohydrate intake [42], among other factors.

## FFA sources and metabolic processes

Fatty acids (FAs) are usually found in organisms in three main esters: TAGs, phospholipids, and cholesterol esters. When FAs are not present in the plasma as esters, they are referred to as non-esterified FAs or FFAs. FAs are mainly taken up as phospholipids and TAGs. During digestion, TAGs are broken down into monoglyceride, diacylglycerol, and FFAs. Short- and medium-chain FAs (≤ 12 carbons) are directly absorbed by intestinal cells. Long-chain FAs (> 12 carbons) are absorbed and re-converted into TAGs and transported via lipoprotein particles such as high-density, low-density, very low-density lipoproteins and chymotrypsin particles [43]. Lipoprotein lipases present on the cell surface cleave TAGs to form FFAs that are taken up by the cells [44]. In the blood, FFAs are mainly carried by serum albumin. In adipose tissues, FFAs are re-esterified to form TAGs, which are stored in adipose droplets of adipocytes and later mobilized through lipolysis, i.e., the hydrolysis of TAGs [18]. In mitochondria, most FFAs undergo β-oxidation to produce acetyl-coenzyme A, and subsequently, acetyl-CoA enters the Krebs cycle to produce energy as adenosine triphosphate (ATP) [18, 45]. FFAs are also important signaling molecules [46]. In addition to dietary intake, FFAs can be synthesized endogenously, which is referred to as de novo adipogenesis, with the main source being (excess) carbohydrates [18, 44].

**Fig. 1:**
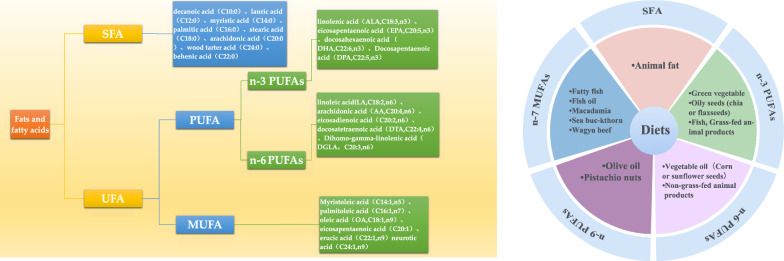
**A** Classification of FFAs; **B** Common sources of dietary intake. **A** present the FFAs contained in SFA, PUFA (n-3/n-6), and MUFA, **B** reflects the common dietary categories of the above fatty acids. FFAs(free fatty acids); SFAs(saturated fatty acids); UFAs(unsaturated fatty acids); MUFAs (monounsaturated fatty acids); PUFAs (polyunsaturated fatty acids)

FFAs are hydrocarbon chains comprising methyl and carboxyl groups at each end. According to the presence or absence and number of carbon–carbon double bonds, FFAs can be categorized as saturated FAs(SFAs, no double bonds), monounsaturated FAs (MUFAs, one double bond), or polyunsaturated FAs (PUFAs, multiple double bonds), and a detailed categorization of the FFA profiles is presented in Fig. 1A. Figure 1B indicates the common sources of dietary intake. Depending on the position of the first double bond at the methyl end of the FFA molecule, PUFAs can be subdivided into n-3, n-6, and n-9 FAs. By measuring and quantifying FFAs, based on the length of the aliphatic tails, short-, medium-, and long-chain FAs have < 6, 6–12, and > 12 carbon atoms, respectively [47]. Commonly used test methods for FFA spectroscopy include gas chromatography- and liquid chromatography-mass spectrometries [48, 49]. FFAs play an important energetic role and are involved in regulating intracellular signaling, cell structure, and lipid mediator production [50]. FFAs can also be converted to sphingolipids and phospholipids, which are involved in cell membrane formation. Among them, ceramide, a type of sphingolipid, is synthesized de novo from SFA (e.g., palmitate) in a multistep enzymatic cascade reaction and is the main scaffold for most complex sphingolipids, which can be broken down into variable FFAs and sphingomyelins. It can affect glucose metabolism by blocking the activation of the anabolic enzyme Akt or protein kinase B (Akt/PKB), inhibiting insulin signaling, and inducing selective insulin resistance, which hinders glucose uptake and storage and promotes hepatic gluconeogenesis [51–53]. Mustaniemi Sanna et al. found that although ceramides, along with clinical risk factors and triglycerides, did not appear to significantly improve the predictive performance of GDM, unfavorable changes in ceramides could indicate the degree of metabolic disturbances in GDM [54].

## Association between dietary FA intake and GDM

GDM development is related to many factors, including the type and amount of FAs consumed in diets. The balance between SFA and PUFA intake is important, and women with GDM reportedly consume significantly less fatty fish representing PUFA but consume a higher proportion of saturated fat [55]. Moreover, SFA intake is positively associated with the development of GDM [56]. Most current studies tend to investigate dietary intake using questionnaires, which makes knowing the amount of each FFA consumed difficult. Humans consume nutrients as part of complex foods that contain varying amounts of nutrients, which may impact the FFA intake from specific sources.

**Fig. 2:**
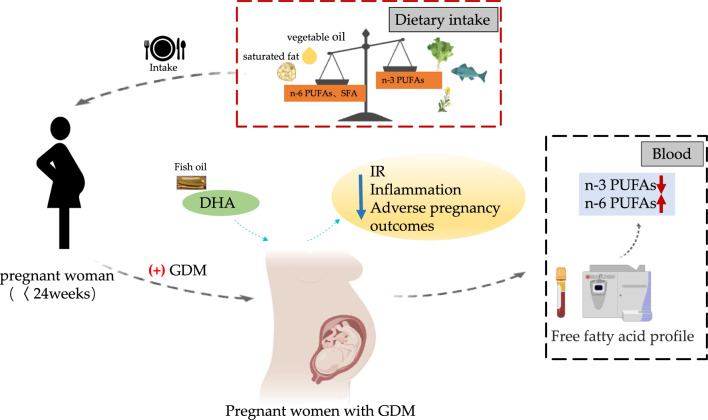
Relationship between dietary intake of FFAs and GDM. Pregnant women with GDM tended to consume more SFA and n-6 PUFAs than n-3 PUFAs, and the results were similar in blood. DHA supplementation is beneficial for pregnant women with GDM. GDM (gestational diabetes mellitus); FFAs(free fatty acids); SFAs(saturated fatty acids); PUFAs (polyunsaturated fatty acids); DHA(docosahexaenoic acid); IR(insulin resistance)

Dietary sources of FFAs play an important role in regulating inflammation. Increased intake of MUFA may be associated with anti-inflammatory effects. One of the most abundant omega-7 MUFA is palmitoleic acid (POA; C16:1 n-7), derived from fatty fish, fish oil, macadamia nuts, sea buckthorn, and Wagyu beef. Oleic acid (OA; C18:1n9) is one of the most abundant MUFA in the human diet [57, 58]. POA activates basal lipolysis and re-esterification, increases lipoprotein lipase (Lpl) activity, regulates n3-PUFA metabolism in membrane phospholipids, and also reduces proinflammatory cytokines to alleviate chronic inflammation. OA may ameliorate inflammation by regu AA metabolism [58]. Studies have shown that dietary supplementation with palmitoleate is beneficial for improving lipid and glucose metabolism and has also been associated with favorable changes in genes that regulate inflammation [58, 59].Yang's previous research has also shown that repeated administration of palmitoleate downregulated the expression of proinflammatory genes, acted as an anti-inflammatory, and blocked glucose or SFA-induced apoptosis of β-cells and improved insulin resistance [60]. Long-chain PUFAs (LC-PUFAs) include two categories: n-3 and n-6 PUFAs. The human body needs to consume some n-3 and n-6 PUFAs from the diet. For example, ALA and LA as essential fatty acids need to be obtained from diets [61]. Typical dietary n-3 sources are oily seeds such as chia or flaxseed, fish, green vegetables, and grass-fed animal products, whereas n-6 is usually derived from vegetable oils such as corn or sunflower seeds, and non-grass-fed animal products (Fig. 1B). Generally, n-6 PUFAs are regarded as pro-inflammatory molecules, whereas n-3 PUFAs (especially eicosapentaenoic acid and docosahexaenoic acid (DHA) are regarded as anti-inflammatory molecules [62]. Maintaining a balance between n-3 and n-6 PUFAs is important for regulating inflammation and immunomodulation. Diets high in n-3 intake can cause reduced cytokine production and lower cardiovascular disease risk factors [63]. However, western diets tend to be low in fiber and rich in total fat, rich in n-6 PUFAs as well as saturated fats, including hydrogenated vegetable fat (HVF, rich in trans fat) and interesterified fat (IF), making the n-6/n-3 PUFA ratio high [64, 65]. Modern diets tend to increase n-6 intake and decrease n-3 intake owing to the increase in processed food consumption, widespread use of hydrogenated vegetable oils, and impact of industrialized agriculture. This has skewed the n-3 to n-6 ratio in favor of n-6, promoting chronic inflammatory diseases [66]. Women with GDM have increased and decreased n-6 and n-3 PUFA blood levels, respectively [67]. In pregnant women with GDM, n-3 PUFA intake is beneficial in alleviating IR and inflammation and decreasing the risk of adverse pregnancy outcomes [68, 69]. Combined supplementation with vitamin D and n-3 PUFAs for 6 weeks improves fasting glucose, very low-density lipoprotein cholesterol, serum triglycerides, and insulin levels in patients with GDM [70]. A higher dietary n-6:n-3 PUFA ratio is associated with higher GDM incidence [71]. Dietary fat-rich fish and oral n-3 supplements are more effective in increasing n-3 PUFA levels [55]. One study found no reduction in the GDM incidence in pregnant women who were supplemented with 800 mg of DHA-enriched fish oil per day [72], and the same results were found in a DHA supplementation study [73]. Thus, DHA appears to be ineffective in preventing GDM but may have a therapeutic role in women with GDM. Pregnant women with GDM may benefit from DHA supplementation, which may be considered in their treatment. MUFA consumption has benefits, and the inclusion of OA (C18:1, n9)-rich olive oil and pistachios in the Mediterranean diet [74, 75] reduces GDM incidence [76]. Olive oil intake reduces inflammatory markers in the placenta and cord blood of pregnant women with GDM [77]. In conclusion, further studies are needed to elucidate the relationship between dietary intake of different FA types and GDM. Figure 2 shows the association between dietary FFA intake and GDM occurrence and development.

## Relationship between FFAs and GDM

### FFAs and IR

IR is the main pathogenesis of GDM, which is mainly characterized by a decrease in sensitivity to insulin [6]. Circulating FFA is one of the most important factors promoting IR and altering insulin secretion [20, 78]. In skeletal muscle, the mechanism by which FFAs cause IR involves protein kinase C activation via diacylglycerol accumulation, leading to decreased tyrosine insulin receptor substrate-1 phosphorylation and phosphatidylinositol-3 kinase activation inhibition [79]. Acute and chronic elevation of FFA induces IR, and Chronic elevation of FFA levels may impede β-cell compensatory responses [80]. A study showed that calculating the FFA index during pregnancy can be used to measure insulin sensitivity [81]. Higher homeostatic model assessment of IR (HOMA-IR) and c-peptide levels can reflect GDM risk. Palmitic acid, stearic acid, AA, dihomo-γ-linolenic acid (DGLA), and DHA positively correlated with HOMA-IR and c-peptide [82], indicating the effect of SFAs, AAs, DGLAs, and DHAs on GDM development. There may be a relationship between inflammation and the development of IR. The saturated fatty acid palmitate promotes the production of pro-inflammatory cytokines associated with insulin resistance in adipose tissue and/or adipocytes [83]. In addition, Maternal obesity may exacerbate the development of IR. POA is a fatty acid with anti-inflammatory and insulin-sensitizing properties, and maternal obesity leads to decreased synthesis of POA by syncytiotrophoblast cells, which can lead to IR and persistent mild inflammation in the mother, placenta, or fetus [84]. Levels of 20-hydroxyeicosatetraenoic acids (20-HETEs), a metabolite of AA, are positively correlated with BMI [85]. Increased levels of cytochrome P450 (CYP) and 20-HETE, which act on AA to exert enzymatic activity, are associated with the development of IR in obese women with GDM [37]. Therefore, maternal obesity should also be of concern when considering the effect of changes in FFAs levels on IR. women with GDM who are predominantly characterized by defective insulin sensitivity have significantly higher FFA [86], also confirming the correlation between FFA and IR.

### FFA levels in pregnant women with GDM

In addition to the measurement of dietary FFA intake, several studies have analyzed the association between blood FFAs and GDM. In healthy pregnancies, serum TG and NEFA levels in mid- to late-gestation, increasing with the number of gestational weeks and peaking just before delivery [87]. The period of greatest increase in FFA and maximum placental FA transport occurs in the last trimester of pregnancy [88]. A trend towards higher plasma FFA levels in patients with GDM was observed [89, 90]. In addition, in a study on the FFA profile of patients with GDM, some differences in FFA levels were found between different gestational periods [55, 91–94]. In Table 1, we present the characteristics of the population of studies and surveys on the FFA spectrum, while the corresponding results are presented in Table 2.

**Table 1:** Studies on population characteristics and blood concentrations of individual FFA species in GDM subjects and controls

References	Number of subjects		Match or not	Population	Period of pregnancy	Sample type	Fasting or not
	Control	GDM					
[92]	43	43	Age and pre-pregnancy BMI	Chinese	8–12w	Serum	Yes
[55]	726	127	Unmatched	Iceland	11–14w	Plasma	No
[91]	138	131	Unmatched	Chinese	24–28w	Serum	Yes
[93]	18	18	BMI	German	27 ± 2w	Plasma	ns
[94]	485	195	Unmatched	Chinese	11–14, 22–28, 32–34w	Serum	ns

**Table 2:** Changes in the concentration of FFAs in the blood of GDM patients compared to controls

Types of FFA	Changes in plasma free fatty acid concentrations in GDM						
	[92]	[55]	[91]	[93]	[94]		
	8-12w	11-14w	24-28w	27 ± 2 w	11-14w	22-28w	32-34w
*Total FFA*		↑		↑			
*Saturated FFA*		↑		↑			
C14:0, myristic acid	↑				↑		↓
C15:0,pentadecanoic acid							
C16:0, palmitic acid	↑		↑		↑		
C17:0,heptadecanoic acid			↑				
C18:0, stearic acid	↑			↑		↑	
C19:0, nonadecylic acid							
C20:0, arachidic acid			↑		↑		
C22:0, behenic acid					↓	↓	↓
C24:0, lignoceric acid					↓	↓	↓
*Monounsaturated FFA*		↑					
C14:1n-9,myristoleic acid							
C16:1n-7,palmitoleic acid	↑			↑			
C16:1n-9,cis-7 hexadecenoic acid			↑		↑		↓
C17:1(cis-10), cis-10-heptadecenoic acid;			↑				
C18:1n-9, oleic acid (OA)			↑		↑	↑	↑
C18:1trans-n-7, vaccenic acid							
C19:1n-9,cis-10 nonadecenoic acid			↑				
C20:1n-9, gondoic acid			↑				
*Polyunsaturated FFA*							
C16:2cis-9,12-hexadecadienoic acid			↑				
PUFA n-3		↑					
C18:3n-3,α-linolenic acid (ALA)	↓?		↑		↑	↑	
C20:5n-3, eicosapentaenoic acid (EPA)						↑	↑
C22:3n-3 docosatrienoic acid			↑				
C22:5n-3, docosapentaenoic acid			↑		↑		
C22:6n-3, docosahexaenoic acid (DHA)				↑	↑		
PUFA n-6		↑					
C18:2n-6, linoleic acid (LA)			↑				
C18:3n-6,γ-linolenic acid (GLA)	↓?		↑		↑		↓
C20:2n-6, eicosadienoic acid			↑		↑		↓
C20:3n-6, dihomo-γ-linolenic acid (DGLA)			↑		↑	↑	↓
C20:4n-6, arachidonic acid (ARA)			↑				
C22:4n-6, adrenic acid							↓
*TransFFA*							
C16:1trans-n-7, trans-palmitoleic acid							
C18:2trans-n-6, linolelaidic acid							
C14:1(trans-9), trans-9-tetradecenoic acid			↑				

Use ↑ to indicate an increase in the concentration of FFAs and ↓ to indicate a decrease

FFAs are altered differently during different gestational periods. Total plasma SFA, MUFA, PUFA n-6, and PUFA n-3 levels increased in pregnant women with GDM in early pregnancy [55]. At mid-pregnancy, with the elevated total FFA, elevated SFA is more pronounced [93]. In Hou et al.’s [91] study, various FFAs were elevated, including OA, ALA, and DGLA, similar to the observations in another study [94]. In early pregnancy, Ma et al. [92] and Zhang et al. [94] reported that myristic and palmitic acids were elevated in pregnant women with GDM, and Ma et al. found that palmitic acid was positively associated with GDM risk. In Zhang et al.'s study, dietary supplementation of ALA and DHA was associated with a high risk of GDM with elevated fasting glucose alone, and the levels of most FFAs, such as DGLA, myristic, palmitoleic, linolenic (γ-LA, GLA), and docosahexaenoic acids, were progressively lower from early to late pregnancy; however, the OA levels were consistently high [94]. In addition, LA levels were reversed [92, 94]. Differences in these data can be interpreted by looking at populations of different races and the controls chosen, whether they match the BMI, and whether fasting is required before blood collection. And the type of sample tested has little effect [95]. Altogether, changes in FFA levels associated with GDM during different gestational periods are complex.

Alterations in the FFA profile of patients with GDM can also affect maternal physiology by altering the levels of FFA transporters, such as increased levels of plasma FA binding protein-4 (FABP4) and other proteins in the serum [96]. Zhang et al. [97] found higher concentrations of FABP4 in GDM women in mid- to late-gestation. FABP4 is positively associated with an increased GDM risk in early and mid-gestation [98].

### Treatment of pregnant women with GDM with FFAs

Currently, the treatment of GDM mainly involves lifestyle interventions and drug therapy. Among the medications are insulin injections or oral glibenclamide and metformin, among others [99]. The traditional dietary treatment for GDM is carbohydrate restriction [100]. However, this approach usually leads to higher fat intake, as protein intake is constant [101]. High-fat diets typically increase serum FFAs and promote insulin resistance [102]. Whereas an appropriate increase in the proportion of carbohydrates and a decrease in the proportion of fat in the diet can still keep blood glucose below current treatment targets and reduce postprandial FFAs [103].

In terms of pharmacological treatment, it has been suggested that increased insulin sensitivity by metformin can lead to a decrease in FFA levels [104]. There is no significant difference in FFA levels between the two modalities of glycemic control in pregnant women with GDM, namely insulin therapy or diet [105].

## FFA and GDM fetal growth and development and other pregnancy outcomes

### Relationship between FFA and GDM fetal growth and development

In addition to glucose, fetal growth is also closely related to lipids [106]. Fasting and 1-h postprandial triglycerides at 16 weeks of gestation are strongly associated with neonatal obesity, more so than maternal glucose [107]. TGs at delivery were independently associated with large for gestational age (LGA) [108]. Placental triglyceride levels and FFA levels in umbilical cord plasma are significantly higher in GDM pregnant women, which may be related to altered placental lipogenesis and fatty acid oxidation protein expression and increased placental transport [109, 110]. It further led to fetal overgrowth. In addition, GDM pregnant women, independently of BMI, altered the lipid profile of the placenta, with elevated levels of both palmitic and stearic acids [93]. Higher maternal FFA levels in GDM reportedly lead to increased fetal birth weights [86], and maternal FFA levels were positively associated with the prevalence of LGA newborns [108, 111]. Emilio Herrera et al. showed a positive correlation between maternal FFA and neonatal weight and adiposity in pregnant women with GDM who had good glycemic control. Maternal dyslipidemia in GDM may promote the transfer of maternal FAs to the fetus, leading to an increase in fetal adipose tissue mass, thereby increasing macrosomia risk [112]. In the study by Fan et al., FFA levels and fetal growth restriction (FGR) were higher in women with GDM than in controls in late pregnancy, and the area under curve value for GDM diagnosis and FGR was 0.84. In addition, a positive correlation was observed between serum FFA and umbilical artery systolic/diastolic ratio (S/D), pulsatility index (PI), and resistance index (RI), reflecting the resistance to blood flow in pregnant women; therefore, fetal growth in a poor intrauterine environment may contribute to FGR development. Moreover, FFA in the serum positively correlated with umbilical artery S/D, PI, and RI values, reflecting blood flow resistance in pregnant women [113]. Studies on the relationship between the FFA profile levels and fetal growth are further needed to utilize FFA concentration or profile levels to help the fetus grow within the appropriate range.

DHA and AA are essential for fetal CNS development and immune system regulation [114]. Reduced maternal-to-fetal DHA transfer in GDM may be associated with impaired placental DHA uptake [115, 116]. Reduced cord blood DHA levels in patients with GDM may lead to reduced plasma DHA levels in neonates with GDM [117, 118]. This outcome affects fetal neurodevelopment in the first 6 months of life [119]. Cord blood DHA levels positively correlate with maternal DHA levels when patients with GDM have good glycemic control [120]. Léveillé et al. also observed no reduction in DHA levels in the umbilical cord blood of pregnant women with GDM treated with diet or medication [121]. It can be seen that effective control of blood glucose levels in GDM patients is beneficial in maintaining fetal DHA levels. To some extent, it favors fetal neurodevelopment. DHA supplementation during pregnancy at 600 mg/d does not improve fetal DHA status [122].

### Association between FFA and other pregnancy outcomes

N-3 and n-6 PUFAs are essential for the structural and functional growth and development of the placenta [123]. In addition to exhibiting anti-inflammatory properties, n-3 FAs have antioxidant potential and play a crucial part in stimulating placenta formation, angiogenesis, and remodeling in early pregnancy [124, 125]. Inadequate maternal LC-PUFA supply and impaired maternal–fetal transfer are associated with adverse pregnancy outcomes, such as preeclampsia, intrauterine growth retardation, and GDM [126, 127]. Neonatal hyperbilirubinemia incidence and neonatal hospitalization in pregnant women with GDM can be reduced by supplementation with n-3 PUFAs [69].

In conclusion, there have been more studies on the impact of altered FFA concentrations on pregnancy outcomes in patients with GDM, but few studies have evaluated the relevance of alterations in different FFA subclasses in patients with GDM to other adverse maternal or neonatal outcome events, and future research could be directed in this direction.

## Discussion

FFAs are derived from various sources, have a high correlation with IR, and are likely to be associated with GDM development. Regarding diet, having a balanced FFA intake is important; however, analyzing a specific FFA is difficult owing to its complexity and various dietary components. Excessive SFA intake can have harmful effects, leading to an increase in inflammation, IR [128], and cardiovascular disease [129]. Before GDM diagnosis, SFA intake was higher than that of PUFAs. When the balance of n-6 PUFAs versus n-3 PUFAs favored the former, a positive correlation was observed with GDM incidence, and preventing GDM by supplementing with n-3 PUFAs has not yet been conclusively demonstrated. Available evidence suggests that n-3 PUFA supplementationis not beneficial in reducing the incidence of maternal pregnancy outcomes such as gestational diabetes and hypertension, but is beneficial for neonatal health, such as reducing the incidence of preterm labor and low birth weight, and increasing birth weight and birth length [130]. Notably, n-3 FA supplementation in pregnant women with GDM is beneficial to reducing IR and inflammation and decreasing adverse pregnancy outcomes. In addition to dietary intake, plasma FFAs reflect FFA levels from various sources, and FFA levels in pregnant women with GDM vary considerably across gestational periods, with the majority being elevated from early to mid-pregnancy and progressively decreasing in late pregnancy. Differences were observed in the results of different studies over the same period, which may be related to factors such as the study populations and whether the relevant indicators were matched. FFAs, palmitic acid, ALA, and DHA are highly correlated with GDM development. In addition, lipocalin is a potent anti-inflammatory/anti-diabetic adipokine [131]. Saturated fatty acids and omega-3 fatty acids are associated with circulating concentrations of lipocalin in healthy humans [132]. Lipocalin is associated with the development of GDM during pregnancy [133]. More studies could be done in the future to evaluate the effects of altered lipocalin and FFAs in patients with GDM.

Lipids are associated with fetal growth and development; FFA levels are closely related to fetal weight, and mechanisms of fetal underweight or overweight require further exploration. Fetal FFA acquisition is regulated by maternal FFA levels and placental mechanisms. DHA is closely related to fetal neurological development, and the reduced DHA transfer to the fetus in patients with GDM may be related to impaired placental uptake, whereas good control of blood glucose promotes the transfer of placental FFAs to an extent, providing sufficient DHA to the fetus. Other adverse pregnancy events are less well-studied. High FFA levels in the postpartum period in women with a previous history of GDM likely contribute to type 2 diabetes mellitus [134, 135]. This may be related to reduced sensitivity to insulin via lipolytic inhibition [136]. Positive recognition of changes in FFAs facilitates a smooth pregnancy course; however, more research and standardization between clinical trials and patient sampling are needed. Finally, appropriate FA intake should be considered before and during pregnancy to optimize maternal and infant outcomes and bring new preventive and therapeutic strategies for GDM.

## Data Availability

Not applicable.

## Abbreviations

GDM: Gestational diabetes mellitus
FFA: Free fatty acid
GWG: Gestational weight gain
IADPSG: International Association of Diabetes and Pregnancy Study Groups
WHO: World Health Organization
ADA: American Diabetes Association
BMI: Body mass index
PIH: Pregnancy-induced hypertension
IR: Insulin resistance
SFAs: Saturated fatty acids
PUFAs: Polyunsaturated fatty acids
ALA: Alpha-linolenic acid
ADA: American Diabetes Association
OGTT: Oral glucose tolerance test
TAGs: Triacylglycerides
FAs: Fatty acids
ATP: Adenosine triphosphate
MUFAs: Monounsaturated fatty acids
UFAs: Unsaturated fatty acids
LPl: Lipoprotein lipase
OA: Oleic acid
POA: Palmitoleic acid
AA: Arachidonic acid
LC-PUFAs: Long-chain polyunsaturated fatty acids
ALA: Alpha-linolenic acid
LA: Linoleic acid
DHA: Docosahexaenoic acid
HVF: Hydrogenated vegetable fat
IF: Interesterified fat
20-HETEs: 20-Hydroxyeicosatetraenoic acids
CYP450: Cytochrome P450
EPA: Eicosapentaenoic acid
GLA: γ-Linolenic acid
DGLA: Dihomo-γ-linolenic acid
ARA: Arachidonic acid
FABP4: FA binding protein-4
LGA: Large for gestational age
FGR: Fetal growth restriction
S/D: Systolic/diastolic
PI: Pulsatility index
RI: Resistance index
HOMA-IR: Homeostatic model assessment of IR
